# Ancestral Vascular Lumen Formation via Basal Cell Surfaces

**DOI:** 10.1371/journal.pone.0004132

**Published:** 2009-01-06

**Authors:** Tomáš Kučera, Boris Strilić, Kathrin Regener, Michael Schubert, Vincent Laudet, Eckhard Lammert

**Affiliations:** 1 Max Planck Institute of Molecular Cell Biology and Genetics (MPI-CBG), Dresden, Germany; 2 Charles University in Prague, The First Faculty of Medicine, Institute of Histology and Embryology, Prague, Czech Republic; 3 Université de Lyon, Institut de Génomique Fonctionnelle de Lyon, Molecular Zoology team, Ecole Normale Supérieure de Lyon, Université Lyon 1, CNRS, INRA, Institut Fédératif 128 Biosciences Gerland Lyon Sud, Lyon, France; 4 Heinrich-Heine-University, Institute of Animal Physiology, Düsseldorf, Germany; Katholieke Universiteit Leuven, Belgium

## Abstract

The cardiovascular system of bilaterians developed from a common ancestor. However, no endothelial cells exist in invertebrates demonstrating that primitive cardiovascular tubes do not require this vertebrate-specific cell type in order to form. This raises the question of how cardiovascular tubes form in invertebrates? Here we discovered that in the invertebrate cephalochordate amphioxus, the basement membranes of endoderm and mesoderm line the lumen of the major vessels, namely aorta and heart. During amphioxus development a laminin-containing extracellular matrix (ECM) was found to fill the space between the basal cell surfaces of endoderm and mesoderm along their anterior-posterior (A-P) axes. Blood cells appear in this ECM-filled tubular space, coincident with the development of a vascular lumen. To get insight into the underlying cellular mechanism, we induced vessels *in vitro* with a cell polarity similar to the vessels of amphioxus. We show that basal cell surfaces can form a vascular lumen filled with ECM, and that phagocytotic blood cells can clear this luminal ECM to generate a patent vascular lumen. Therefore, our experiments suggest a mechanism of blood vessel formation via basal cell surfaces in amphioxus and possibly in other invertebrates that do not have any endothelial cells. In addition, a comparison between amphioxus and mouse shows that endothelial cells physically separate the basement membranes from the vascular lumen, suggesting that endothelial cells create cardiovascular tubes with a cell polarity of epithelial tubes in vertebrates and mammals.

## Introduction

It has been suggested that the cardiovascular system of bilaterians evolved from a common ancestor [1]–[3]. This is because the heart and major blood vessels develop as tubes along the anterior-posterior (A-P) axes in both vertebrates and invertebrates [4], [5]. In addition, several genes have been identified in both vertebrates and invertebrates that have similar expression domains and functions in cardiovascular development. For example, the homeotic gene *tinman* and its homologue *NKX2.5* are expressed in cardiac mesoderm in Drosophila and mouse, respectively, and these genes are required for proper cardiac development in both animals [1], [5]. Despite conservation of several genes involved in cardiovascular development, new features evolved in the vertebrates. For example, in vertebrates, endothelial cells line the lumen of the heart and of all blood vessels [6]. In contrast, in invertebrates, endothelial cells either are not present or do not form a continuous vascular wall [3], showing that endothelial cells are not a conserved feature of cardiovascular tubes. Therefore, in order to understand the ancestral and conserved part of cardiovascular tube formation, we investigated developing vessels in the invertebrate amphioxus and compared these vessels with the homologous ones in mouse. We used the cephalochordate amphioxus, *Branchiostoma lanceolatum*, because its body plan is similar to the one of vertebrates [7], [8], and because it forms a monophyletic clade with vertebrates and urochordates [9].

## Results

### Localization of basement membrane in amphioxus and mouse vessels

**Figure 1:**
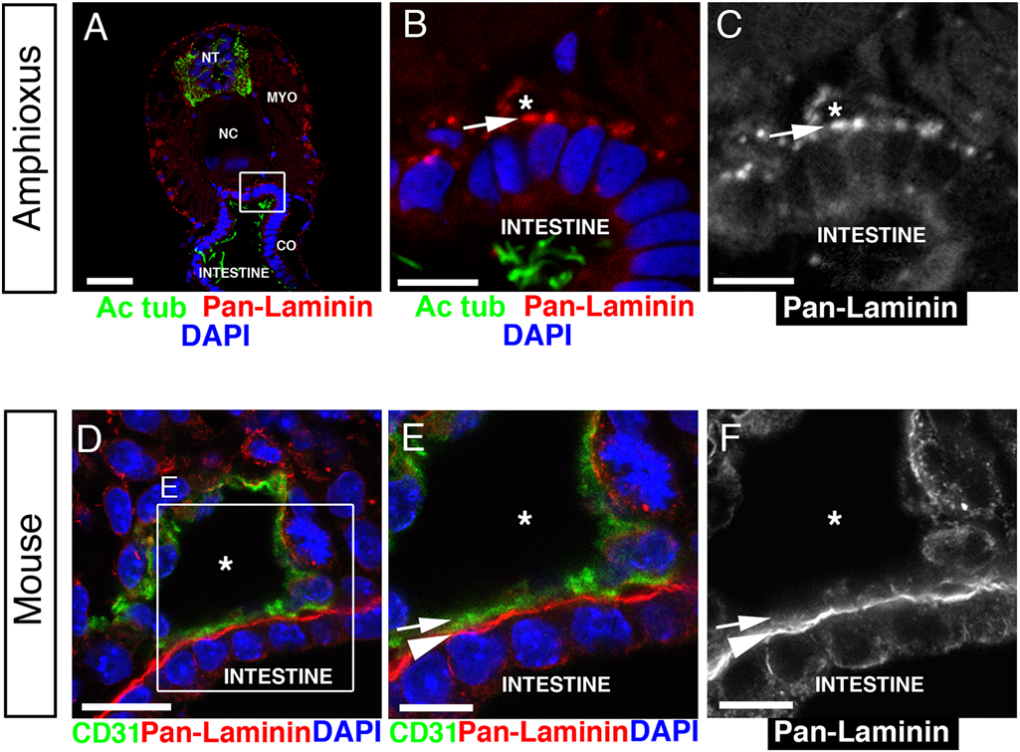
Basal cell surfaces line the vascular lumen in amphioxus. (A) Confocal image of a transverse section through an anterior part of juvenile amphioxus stained for laminin (red) to localize basement membranes, and acetylated tubulin (green) to localize apical cilia. Cell nuclei are stained with DAPI (blue). NT, neural tube. NC, notochord. MYO, myomere. CO, coelom. Scale bar, 20 µm. (B) Higher magnification of the boxed area outlined in (A) shows laminin (red) lining the lumen (asterisk) of the amphioxus aorta. The apical cilia (green) of the intestinal cells project into the intestinal lumen. Nuclei are labeled with DAPI (blue). Scale bar, 5 µm. (C) The red channel from (B) shows laminin staining (arrow) in the lumen (asterisk) of the amphioxus aorta. Scale bar, 5 µm. (D) Confocal image of a transverse section through an E9.0 mouse embryo shows a dorsal aorta with a lumen (asterisk). Endothelial cells stained for CD31 (green) line the vascular lumen and separate it from the laminin-containing basement membrane (red) of the intestinal epithelium. Nuclei are labeled with DAPI (blue). Scale bar, 20 µm. (E) Higher magnification of the boxed area outlined in (D) shows endothelial cells (green) lining the aortic lumen (asterisk) with their laminin-free luminal cell surface (arrow). The abluminal cell surface (arrowhead) is adjacent to the laminin-containing basement membrane of the intestinal epithelium (red). DAPI (blue). Scale bar, 10 µm. (F) The red channel from (E) shows laminin staining on the abluminal cell surface (arrowhead) of the mouse aorta. Scale bar, 10 µm.

As shown in Fig. 1, both the amphioxus aorta (Fig. 1A–1C, asterisk) and the larger mouse aorta (Fig. 1D–1F, asterisk) are located on top of, or dorsal to, the adjacent intestine. Based on previous morphological studies, an electron dense layer, morphologically similar to a basement membrane, has been observed on the luminal side of invertebrate blood vessels [3], [10]–[12]. However, it remained to be shown whether invertebrate vessels were lined by a real basement membrane or, alternatively, by an apical ECM that is morphologically similar, but molecularly different from a basement membrane [13]–[16].

To clarify this issue, we localized the basement membrane protein laminin on transverse sections through amphioxus (Fig. 1A–1C). As shown in Fig. 1B, this basement membrane protein (shown in red color) surrounds the lumen of the aorta in amphioxus (asterisk). By contrast, in mouse, endothelial cells (shown in green color in Fig. 1D and 1E) surround the aortic lumen (asterisk in Fig. 1E) and separate it from the laminin-containing basement membrane (shown in red color in Fig. 1E).

We also localized acetylated tubulin (Ac tub) as an apical marker that labels primary cilia in amphioxus (as shown in green color in Fig. 1A and 1B). We found that this apical marker was exclusively localized to the surface of the intestinal epithelium that faced the gut lumen (lower part of Fig. 1A and 1B). Since we observed laminin, but not acetylated tubulin, in the aortic lumen (asterisk in Fig. 1B), we conclude that the intestinal epithelium is polarized. It faces the gut lumen with its apical cell surface harboring cilia (green color in Fig. 1B) and the aortic lumen with its basal cell surface and basement membrane (arrow in Fig. 1B). Therefore, even though in both animals the aortae form on the basement membrane of the intestinal epithelium (Fig. 1B and 1E), endothelial cells introduced a change in mouse. In amphioxus, the basement membrane of the intestinal epithelium directly lines the vascular lumen (arrow in Fig. 1B and 1C), whereas in mouse, endothelial cells (shown in green color in Fig. 1E) separate the intestinal basement membrane (arrowhead in Fig. 1E) from the aortic lumen (asterisk in Fig. 1E).

### Induction of vessels filled with luminal ECM

**Figure 2:**
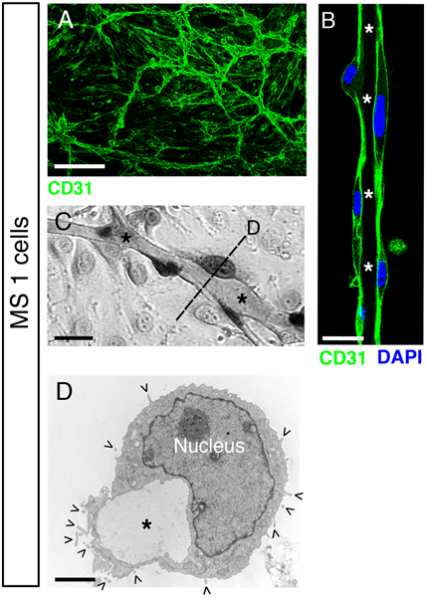
Matrigel overlay induces an invertebrate vascular morphology in MS1 endothelial cells. (A) Confocal image of a tubular network formed by Mile Sven 1 (MS1) cells upon Matrigel overlay. Vessels are stained for CD31 (green). Scale bar, 200 µm. (B) Confocal image at higher magnification of a vessel stained for CD31 (green) and cell nuclei (DAPI, blue). The lumen is labeled with asterisks. Scale bar, 10 µm. (C) Phase-contrast image of a hematoxylin-eosin stained vessel with a lumen (asterisks). Scale bar, 10 µm. (D) Electron micrograph showing a cross-section through a vessel with a lumen (asterisk). Microvilli (ˆ), characteristic of apical cell surfaces, are exclusively localized on the abluminal plasma membrane, whereas the luminal plasma membrane has a smooth appearance, characteristic of basal cell surfaces. Scale bar, 2 µm.

Since a laminin-containing basement membrane lines the vessels in amphioxus, we asked whether Matrigel, a reconstituted basement membrane, or basal ECM, was able to induce similar kinds of vessels *in vitro* (Fig. 2). Matrigel induces tube-like structures in several cell types, including endothelial cells and smooth muscle cells [17], [18], and we used an immortalized endothelial cell line, Mile Sven 1 (MS1), for most experiments described in this study. A branched network of vascular tubes formed in 24–48 hrs after Matrigel overlay (Fig. 2A). The average length of MS1 tubes between intersections was 112±47 µm, and the average lumen width was 3.8±2.5 µm (n = 20). As shown by light microscopy (Fig. 2A to 2C) and electron microscopy (Fig. 2D), the Matrigel-induced multicellular vessels had a visible lumen (asterisks). Importantly, the luminal cell surface was relatively smooth (Fig. 2D), indicative of a basal cell surface, whereas the abluminal cell surface possessed microvilli (open arrowheads), indicative of an apical cell surface. Finally, we detected an electron-dense material inside the vessel lumen (asterisk in Fig. 2D), which resembled the electron-dense material observed in developing vessels in amphioxus [19]. Therefore, our data show that MS1 vascular tubes induced by Matrigel overlay do not reflect vertebrate blood vessels, but instead resemble the vessels observed in invertebrates, such as in amphioxus.

### Molecular composition of the luminal ECM in vessels formed by MS1 cells

**Figure 3:**
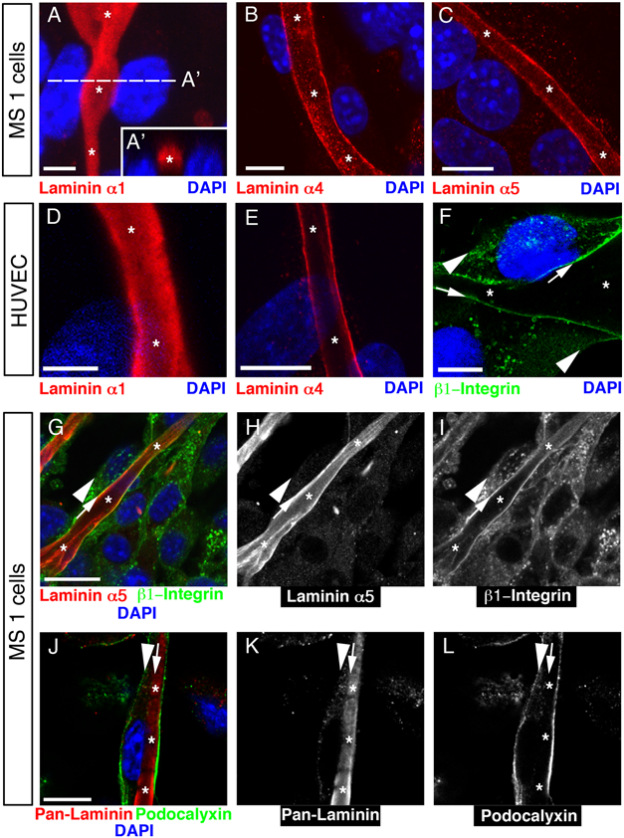
MS1 cells and HUVEC can use their basal cell surfaces to form a vascular lumen around basal ECM. (A) Projection of a confocal z-stack through a vessel formed by MS1 cells shows that the lumen (asterisks) is filled with the Matrigel-derived laminin α1 chain (red). (A′) An optical cross-section is shown at the location indicated by a dashed line in (A). Nuclei (DAPI, blue). Scale bar, 5 µm. (B, C) Confocal images show the endothelial cell-specific (B) laminin α4 chain (red) and (C) laminin α5 chain (red) on the luminal cell surface of MS1 cells. Nuclei (DAPI, blue). Scale bars, 10 µm. (D–F) Confocal images of vascular tubes with a lumen (asterisk) formed by HUVEC 48 hrs after Matrigel overlay. (D) The lumen stains for Matrigel-derived laminin α1 chain (red) and (E) endothelial cell-derived laminin α4 chain (red). Nuclei are labeled with DAPI (blue). Scale bars, 5 µm in (D) and 10 µm in (E). (F) A HUVEC tube stained for β1-integrin (green), which is present on the luminal (arrows), rather than on the abluminal plasma membrane (arrowheads). Nuclei are labeled with DAPI (blue). Scale bar, 10 µm. (G) Confocal image of a tube formed by MS1 cells showing laminin α5 chain (red) and its β1-integrin receptor (green) on the luminal cell surface (arrow), but not on the abluminal cell surface (arrowhead). Nuclei (DAPI, blue). Scale bar, 20 µm. (H) The red channel from (G) shows laminin α5 chain on the luminal cell surface (arrow). (I) The green channel from (G) shows that β1-integrin is localized on the luminal cell surface (arrow). (J) Confocal image of a tube formed by MS1 cells shows that the apical marker podocalyxin (green) is localized on the abluminal cell surface (arrowhead), whereas the basement membrane protein laminin (red) is located inside the vascular lumen (asterisks). Nuclei (DAPI, blue). Scale bar, 10 µm. (K) The red channel from (J) shows laminin inside the lumen (asterisks). (L) The green channel from (J) shows localization of podocalyxin on the abluminal cell surface (arrowhead).

**Figure 4:**
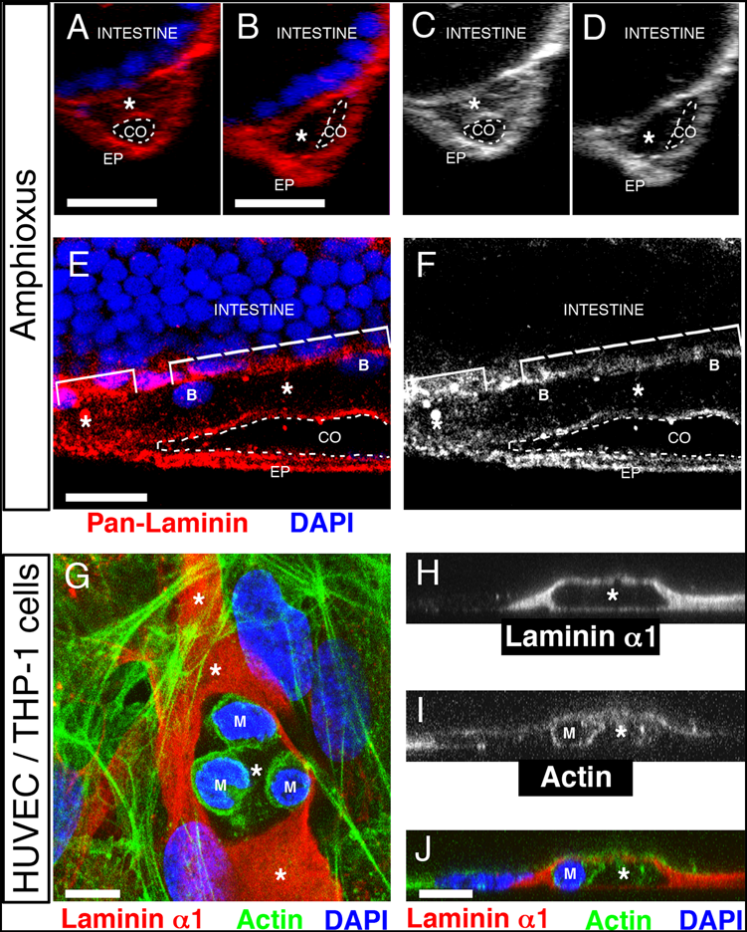
Phagocytotic blood cells generate a patent vascular lumen. (A–F) Confocal images of 14-day old amphioxus larvae stained for laminin (red) and nuclei (DAPI, blue). Asterisks label the lumen of the subintestinal vessel. CO, coelom (dashed line); EP, epidermis. Scale bars, 10 µm. (A–D) Transverse sections. (A) An area of the vascular lumen (asterisk) that is filled with laminin (red). (B) An area of patent vascular lumen (asterisk) that is only lined by laminin. (C) Red channel from (A) showing an area filled with laminin (asterisk). (D) Red channel from (B) showing an area of a patent vascular lumen (asterisk). (E–F) Longitudinal sections. (E) Less laminin (red) is found in the lumen of the subintestinal vessel that contains blood cells (B) (dashed bracket), compared to the lumen that is without any blood cells (small bracket). (F) Red channel from (E) showing less laminin on the right side of the vessel where blood cells are found. (G) Confocal image of a vessel formed by HUVEC cells in the presence of a macrophage/monocyte cell line THP-1 (M). Macrophages (M) are found in a part of the vascular lumen that is without any laminin filling (red). Scale bar 5 µm. (H–J) A cross-section through a vessel shows a macrophage (M) inside a patent vascular lumen (asterisk) that is lined by, but not filled with laminin. (H) Laminin α1 chain is absent from the lumen (asterisk) that contains (I, J) a macrophage (M). (J) The composite image shows the localization of laminin α1 chain (red), actin (green) and nuclei (blue). Scale bar, 10 µm.

Next we characterized the composition of the luminal ECM localized inside vessels formed by MS1 cells after Matrigel overlay. Laminin-α1 chain is a basement membrane protein present in Matrigel [20], and endothelial cells do not produce it [21]. In contrast, endothelial cells produce laminin-α4 and -α5 chains, which are not present in Matrigel [21], [22]. Using laminin-α-chain specific antibodies, we found that the laminin-α1 chain was uniformly present within the vascular lumen (Fig. 3A), whereas the endothelial cell-produced laminin-α4 and -α5 chains were found close to their luminal cell surface (Fig. 3B and 3C). These data show that Matrigel fills the vascular lumen and suggest that it induces MS1 cells to deposit basement membrane proteins towards the developing vascular lumen. A similar situation was found after overlaying human umbilical vein endothelial cells (HUVEC) with Matrigel (Fig. 3D–3F). Based on the localization of basement membrane proteins, the resulting vessels molecularly resemble the vessels observed in amphioxus (compare Fig. 3B and 3C with Fig. 4E).

### Lumen formation via basal cell surfaces in Matrigel

Our data suggest that basal cell surfaces and their ECM are the ancestral components of blood vessels. To provide evidence for vascular lumen formation via basal cell surfaces, we analyzed the localization of apical and basal cell surface markers in vascular tubes formed by MS1 cells. In most epithelial tubes (or cysts), β1-integrin is preferentially localized to the abluminal or basal cell surface next to the basement membrane [23], whereas podocalyxin (gp135) is exclusively localized to the luminal or apical cell surface [24].

In stark contrast to the localization of apical and basal proteins in epithelial tubes, we observed strong β1-integrin expression on the luminal cell surface (arrow in Fig. 3I) rather than on the abluminal cell surface of MS1 cells (arrowhead in Fig. 3I). Conversely, podocalyxin (gp135) was expressed on the abluminal cell surface (arrowhead in Fig. 3J and 3L) rather than on the luminal cell surface (arrow in Fig. 3J and 3L). Given the abundance of β1-integrin on the basal cell surface of tube-forming cells (Fig. 3I), we asked whether integrins are involved in this type of tube formation. Thus, we applied a function-blocking antibody to interfere with β1-integrin signaling and quantified the numbers of open and closed tubes after 2 days of culture. In assays of MS1 cells treated with β1-integrin function blocking antibody, the frequency of open tubes (Fig. S1) was four times higher compared with control antibody treatment (Fig. S1). These data show that β1-integrin is involved in tube formation of MS1 cells upon Matrigel overlay.

These findings demonstrate that the basal cell surface of MS1 cells is capable of forming a vascular lumen. This atypical tube formation may have been due to unique properties of MS1 endothelial cells used in this assay. Therefore, we performed the Matrigel overlay assay with primary human umbilical vein endothelial cells (HUVEC), which are frequently used for vascular tube formation studies [25], [26]. Similar to MS1 cells, HUVEC formed tubes, which contained both exogenous (Fig. 3D) and endogenous (Fig. 3E) laminins inside the lumen. In addition, the luminal plasma membrane of HUVEC had a more intense β1-integrin expression (arrows in Fig. 3F) compared to the abluminal membrane (arrowheads in Fig. 3F).

To test whether Matrigel as a basal ECM was responsible for inducing tubes via basal cell surfaces, we used an assay in which HUVEC cells form lumenized vascular sprouts in fibrin gel [25]. When we analyzed the localization of laminin in these sprouts we found it on the abluminal cell surface (arrowhead in Fig. S2A, B). The lumen (asterisk in Fig. S2A and S2B) as well as the luminal cell surface (arrows in Fig. S2A) was laminin-free. In addition, we performed an EM analysis of HUVEC vascular tubes formed in fibrin gel and we detected a basement membrane on the abluminal surface of HUVEC (Fig. S2C and S2D).

Therefore, these results indicate that Matrigel ECM, rather than fibrin, can induce endothelial cells to form tubes with intraluminal ECM.

### Blood cells-mediated removal of luminal ECM

Blood vessels of early amphioxus larvae are filled with an electron-dense material [19], [27]. In adult amphioxus, however, this material has disappeared [19]. Since blood cells in amphioxus are phagocytotic and share functional similarities to macrophages in vertebrates [28], [29], we investigated whether an ECM-free lumen coincided with the localization of blood cells. To this end, we focused on the subintestinal vessel, which is the heart and the largest vessel in this animal [30]. As shown in Fig. 4A to 4D, the subintestinal vascular lumen contained areas completely filled with laminin (asterisks in Fig. 4A and 4C) as well as areas, which were only lined by laminin (Fig. 4B and 4D). Importantly, the localization of blood cells (B) inside the subintestinal vessel coincided with the presence of a patent vascular lumen (dashed bracket in Fig. 4E and 4F). In contrast, areas with no blood cells were filled with laminin-containing ECM (small bracket in Fig. 4E and 4F).

To test whether phagocytotic cells were able to remove luminal ECM, Matrigel overlay was used to induce HUVEC vessels in the presence of the monocyte/macrophage cell line THP-1 (labeled with “M” in Fig. 4G–4J). Following this co-culture we detected an absence of luminal ECM in regions, where these blood cells were incorporated into the vascular lumen (Fig. 4G, and a cross-section through a vascular lumen in Fig. 4H–4J). We also detected cleared areas of lumen, which these cells appeared to have left (data not shown). There are several possibilities how macrophages can remove basal ECM: phagocytosis, extracellular proteolysis, or both phagocytosis and extracellular proteolysis. To determine the mechanism of macrophage-mediated Matrigel removal we used DQ collagen IV as a substrate that becomes fluorescent upon proteolytic cleavage. Collagen IV is a component of Matrigel and can be detected intraluminaly in tubes formed by HUVEC (Fig. S3A, S3B). We incubated THP-1 monocyte/macrophage cells with Matrigel containing DQ collagen IV. After 24 hours we analyzed the cells microscopically and we observed the fluorescent cleavage product inside THP-1 cells (arrows in Fig. S3C, S3D) as well as extracellularly around THP-1 cells (arrowheads in Fig. S3C, S3D). This finding demonstrates that THP-1 macrophages digest the Matrigel by extracellular proteolysis as well as by phagocytosis. The results therefore suggest that phagocytotic blood cells are involved in the formation of a patent vascular lumen during vessel formation via basal cell surfaces.

## Discussion

**Figure 5:**
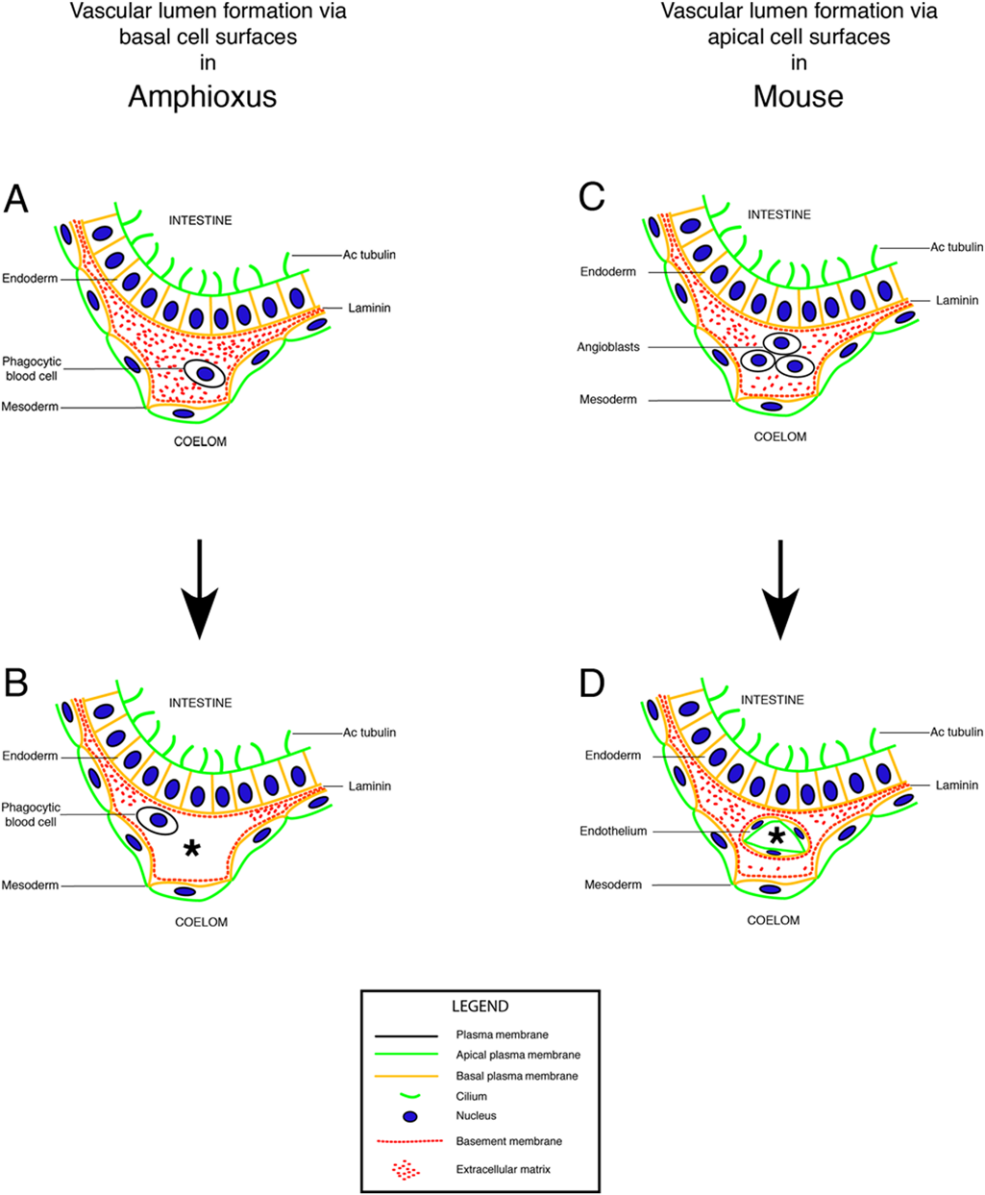
Formation of a basal vascular lumen in invertebrates and creation of an apical vascular lumen in vertebrates. Hypothesis: In invertebrates, basal cell surfaces and basement membrane line the vascular lumen. In vertebrates, by contrast, evolution of endothelial cells led to the formation of blood vessels with apical cell surfaces lining the vascular lumen. This physically separated the basement membranes from blood. (A, B) The first vascular lumen (asterisk) in the invertebrate chordate amphioxus (*Branchiostoma lanceolatum*) forms between the basal cell surfaces of endoderm (e.g. intestinal epithelium) and mesoderm (e.g. coelomic mesothelium). (A) The blood vessel is filled with basally deposited ECM that contains laminin. (B) Phagocytotic blood cells remove this ECM to create a patent vascular lumen (asterisk) between the basal cell surfaces. (C, D) The first vascular lumen (asterisk) inside the mouse (*Mus musculus*) also forms between the basal cell surfaces of endoderm (e.g. intestinal epithelium) and mesoderm (e.g. coelomic mesothelium). (C) However, endothelial cells, or angioblasts, develop from the mesoderm and populate the ECM between the basal cell surfaces. (D) Finally, endothelial cells generate a blood vessel and separate the vascular lumen (asterisk) from the surrounding basement membranes.

Our study provides the first model of vessel formation in amphioxus and possibly in other invertebrates (Fig. 5A and 5B). Invertebrates account for more than 99% of living species, and a cardiovascular system already occurred in a bilaterian ancestor, most likely prior to the divergence of deuterostomes and protostomes [1]. The finding that the Drosophila heart is induced by expression of a homeotic gene (tinman), which was later found to have a homolog (NKX2.5) with a similar role in mouse, supports the notion that the cardiovascular system of most bilaterians developed from a common ancestor. To understand the ancestral mechanism of vessel formation, we investigated the invertebrate cephalochordate amphioxus and compared its developing vessels with the homologous ones in mouse (Fig. 5). We showed that vessel formation is initiated between the basal cell surfaces of endoderm and mesoderm (Fig. 5A). For instance, the heart, or subintestinal vessel, develops between the basal cell surfaces of the intestinal epithelium and the mesothelium that face with their apical cell surfaces the gut tube (upper part of Fig. 5A) and the coelomic cavity (lower part of Fig. 5A), respectively. Therefore, the vascular lumen must form between the basal cell surfaces of these two polarized epithelial cell layers. The vessels are initially filled with a laminin-containing ECM that we term “basal ECM” (Fig. 5A) to distinguish it from apical ECM.

Our *in vitro* experiments show that Matrigel, a reconstituted basement membrane or basal ECM, is sufficient for inducing vessel formation via basal cell surfaces. To our knowledge, this is the first demonstration that the lumen of multicellular tubes can form via basal cell surfaces with the apical cell surface oriented abluminally. Since the endoderm is the first polarized epithelium to form inside the developing embryo [31], its basement membrane may be the initial site of vessel formation. Subsequently, mesoderm-derived cells contribute to vessel formation and provide basal ECM and blood cells. In Drosophila, most blood cells are phagocytotic and produce as well as degrade ECM [2], [29]. In support of a role of blood cells in the formation of a vascular lumen, incorporation of a macrophage/monocyte cell line into developing Matrigel-induced vessels led to the development of an ECM-free vascular lumen (asterisk in Fig. 5B). Recently, two reports were published on the mechanism of lumen formation in the dorsal vessel of Drosophila [32], [33] that reveals similarities as well as differences compared to the mechanism of vascular tube formation in amphioxus that we described here. In Drosophila, the lumen is formed via repulsion of distinct plasma membrane domains having basal characteristics, showing that the dorsal vessel also forms via basal cell surfaces. In contrast to amphioxus, however, where the abluminal plasma membrane is apical, the abluminal cardioblast cell surface in Drosophila also has a basal character.

Interestingly, the mechanism of vessel formation via basal cell surfaces in invertebrates may also help to explain vasculogenic mimicry by which certain malignant cancer cells form primitive vascular channels within tumor tissue. Similar to the invertebrate blood vessels, laminin-containing ECM lines these vessels within the tumor [34]. Therefore, our findings warrant further research to find out whether cancer cells resume an ancestral mechanism of vessel formation as we described here (Fig. 5A and 5B).

Similar to amphioxus, blood vessel formation in mouse often happens on, or between, basal cell surfaces. However, in the case of the heart and aortae, mesoderm-derived endothelial cells develop between the basal cell surfaces of endoderm and mesoderm (Fig. 5C). When these vertebrate-specific cells form a vessel, they separate the basement membranes from the developing vascular lumen (Fig. 5D). Therefore, our data suggest that the evolution of endothelial cells in vertebrates led to the creation of blood vessels with a morphology and cell polarity similar to epithelial tubes (compare Fig. 5D with Fig. 5B). Moreover, it is likely that this event may have paved the way for a more modular cardiovascular and hematopoietic system in vertebrates and mammals.

## Materials and Methods

### Antibodies and reagents

The following primary antibodies were used: rat anti-mouse CD31 (PECAM-1) (BD Pharmingen), rat anti-mouse β1-integrin and rabbit anti-mouse collagen IV (Chemicon), mouse anti-β1 integrin (Santa Cruz), rabbit anti-laminin-111 (EHS) (Sigma), rabbit anti-laminin chain α1 [35], α4, and α5 [21], goat anti-podocalyxin (R&D), mouse anti-acetylated tubulin (Sigma). Hamster anti-rat CD29 (Ha2/5) and hamster IgM (both BD Pharmingen) were used as β1 integrin blocking and control antibody, respectively. DQ™ Collagen type IV was purchased from Invitrogen. Matrigel™ (BD Biosciences), recombinant murine vascular endothelial growth factor (VEGF A -164) and recombinant human fibroblast growth factor-b (FGF-b) (Biomol) were used in vascular tube formation assays.

### Cell lines and cell culture

Mouse microvascular endothelial cells (MS1) (ATCC) were grown in Dulbecco's modified Eagle's medium/high glucose (DMEM) supplemented with 5% fetal calf serum, 100 U ml^−1^ penicillin, 100 µg ml^−1^ streptomycin and 4 mM L-glutamine (Gibco). Human umbilical vein endothelial cells (HUVEC) were used at passage 2–6, and were grown in EGM-2 media (Lonza). THP-1 macrophage/monocyte cells (ECACC) were grown in RPMI 1640 (PAA), 2 mM glutamine and 10% fetal bovine serum (Gibco). Human skin fibroblasts (Detroit 551) ATTC – CCL-110 were kept in Eagle's Minimum Essential Medium with 10% fetal bovine serum (Gibco).

### Vascular tube formation assays with Matrigel

A modification of a previously published assay [17] was used. In brief, MS1 cells or HUVEC were plated either on 8-well chamber slides (BD Falcon) or on 35 mm glass-in-bottom Petri dishes (MaTek Corporation). After 6–8 hrs, the cells were washed with appropriate serum-free media and overlaid with Matrigel™ diluted 1∶5 in serum-free media. After gel formation over 30 min at 37°C in the incubator, the gels were overlaid with 0.5 ml of culture media (in an 8-well chambered slide) or 3 ml of culture media (in a Petri dish). For MS1 cells the culture medium was supplemented with 40 ng/ml VEGF and 40 ng/ml FGF-b.

### Electron microscopy

Cells were fixed with 2.5% glutaraldehyde in 0.1 M Na-cacodylate buffer for 1 h. Subsequently, cells were washed and stained with OsO_4_ and uranyl acetate, dehydrated in graded solutions of ethanol, and embedded in Epon resin. Ultrathin sections (70 nm) were contrasted by using lead citrate and uranyl acetate and observed with Philips EM400T or Tecnai 12 Biotwin (FEI) electron microscopes. Images were taken with Mega View II (SIS) or TemCam F214A (TVIPS) digital camera, respectively.

### Immunocytochemistry and immunohistochemistry

Cells and tissues were fixed with 4% paraformaldehyde in PBS (pH = 7.4) and used for immunocytochemistry and -histochemistry as recently described [36]. In brief, unspecific binding was blocked using 1% BSA, 5% normal serum, 0.1% Tween 20 or Triton-X-100 in PBS ( = blocking buffer) for 1 h. Primary antibodies were applied overnight at 4°C and, after washing, secondary antibodies were applied for 1 h at room temperature. Both antibodies were diluted in blocking buffer. Confocal imaging was performed using a LSM 510 laser scanning confocal system equipped with LSM 510 software (Zeiss). Plan-Apochromat 63×/ 1.4 Oil DIC, 40×/ 1.2 Apo W Corr and 10×/ 0.3NA objectives (Zeiss) were used.

### Immunohistochemical analysis of amphioxus

Juvenile specimens of amphioxus (*Branchiostoma lanceolatum*) fixed in formalin were obtained from Biologische Anstalt Helgoland of the Alfred-Wegener-Institute. Specimens were processed for immunohistochemistry on cryosections as described above.

Whole-mount immunolabeling was performed on 14 day-old larvae fixed in 4% paraformaldehyde and preserved in 70% ethanol. Larvae were rehydrated and permeabilized using 0.2% Triton-X-100. Incubations with antibodies were performed for 24 hrs at 37°C.

### Vascular tube formation assay in co-culture with THP-1 cells

Vessel formation by HUVEC was induced in 24-well plates with coverslips at the bottom as described above, but with the following modification: THP-1 cells were mixed with Matrigel (10.000 cells/well); HUVEC cells were overlaid with 180 µl of this mixture. Co-cultures were kept for 72 hrs, fixed and labeled for laminin α-1 chain and actin.

### Blocking of β1-integrin in Matrigel overlay tube formation assay

MS1 cells were plated into 24-well plates that contained glass coverslips at the bottom of each well. After 10 hrs the cells were pretreated for 30 min with blocking or control antibody at a concentration 10 µg/ml in culture media or with media alone. Cells were washed with serum-free media and were overlaid with Matrigel™ diluted 1∶5 in serum-free media without or with antibodies to obtain 10 µg/ml concentration. After gel formation over 30 min at 37°C in the incubator, the gels were overlaid with media without or with antibodies (10 µg/ml). After 48 hrs of culture, the cells were fixed and stained for CD31 to label the endothelial plasma membrane. Confocal z-stacks of tubes (n = 9–12 per assay from 3 different assays) were examined for the presence of openings. Tubes containing openings bigger than 1 µm were quantified as open tubes. Statistical difference was determined using Student's t-test.

### Vascular tube formation assay using HUVEC in fibrin gel

A modification of an already published assay was used [25]. HUVEC on collagen-coated beads were embedded in the fibrin gel in 35 mm glass-in-bottom Petri dishes (MaTek Corporation). Human skin fibroblasts (100,000/ dish) were added on top of the gel in 3 ml of full EGM-2 media. After 12 days the lumenized vascular sprouts formed and the cells were processed for immunocytochemistry and electron microscopy.

### Visualization of matrix proteolysis by THP-1 cells using DQ™ collagen IV

THP-1 cells were mixed with Matrigel diluted 1∶5 with EBM-2 media containing 20 µg/ml DQ™ collagen IV, fluorescein conjugate (Invitrogen) and the 200 µl of the mixture was pipetted into glass-in-bottom Petri dishes. After 30 min at 37°C Matrigel gelled and 3 ml of EGM-2 media were added to each dish. After 24 hrs the cells were fixed, stained with phalloidine-rhodamine and DAPI.

## Supporting Information

Figure S1 Impaired vascular tube formation of MS1 cells after treatment with β1-integrin blocking antibody. Quantification of open MS1 endothelial tubes in Matrigel overlay assays treated with control and β1-integrin blocking antibodies. N = 3 tube formation assays. *p<0.01. All values are means±SD.
(0.60 MB TIF)

Figure S2 Localization of basement membrane and laminin in vascular tubes formed by HUVEC in fibrin gel. (A–B) Confocal images of a vascular tube formed by HUVEC in the fibrin gel stained for laminin (green). (A) A single plane of a z-stack shows laminin localized on the abluminal cell surface (arrowhead) facing the fibrin gel. The luminal plasma membrane (arrow) outlined by a dashed line surrounds the lumen (asterisk), which is free of laminin. Scale bar, 10 µm. (B) An optical cross-section is shown at the location indicated by a dashed line in (A). Nuclei (DAPI, blue). Scale bar, 10 µm. (C) Electron micrograph showing a cross-section through a vascular tube with a lumen (asterisk) formed by HUVEC in fibrin gel. Scale bar, 2 µm. (D) A higher magnification of a boxed area outlined in (C) showing the abluminal cell surface covered by a basement membrane (arrowheads). Scale bar, 500 nm.
(4.70 MB TIF)

Figure S3 Mechanism of Matrigel removal by THP-1 macrophages. (A, B) Confocal images showing a vascular tube formed by HUVEC. (A) The tube is stained for collagen IV (red) and β1-integrin (green). Collagen IV fills the lumen (asterisk) of a tube. Nuclei (DAPI, blue). (B) The red channel from (A) shows the tube stained for collagen IV only. Scale bar, 10 µm. (C, D) Confocal images showing THP-1 cells in Matrigel mixed with DQ collagen IV. (C) The green channel fluorescence corresponds to the proteolytically degraded DQ collagen IV. THP-1 cells are stained for actin (red). The cleavage product can be found both intracellulary (arrow) and extracellulary (arrowhead) adjacent to THP-1 cell surfaces. Nuclei (DAPI, blue). (D) The green channel from (C) showing proteolytically cleaved DQ collagen IV only. Scale bar, 10 µm.
(2.33 MB TIF)
