# Supplementary figures and images for: Ancestral Vascular Lumen Formation via Basal Cell Surfaces
Source: PLoS One. 2009 Jan 6;4(1):e4132. doi: 10.1371/journal.pone.0004132 (PMC2607016; doi:10.1371/journal.pone.0004132)

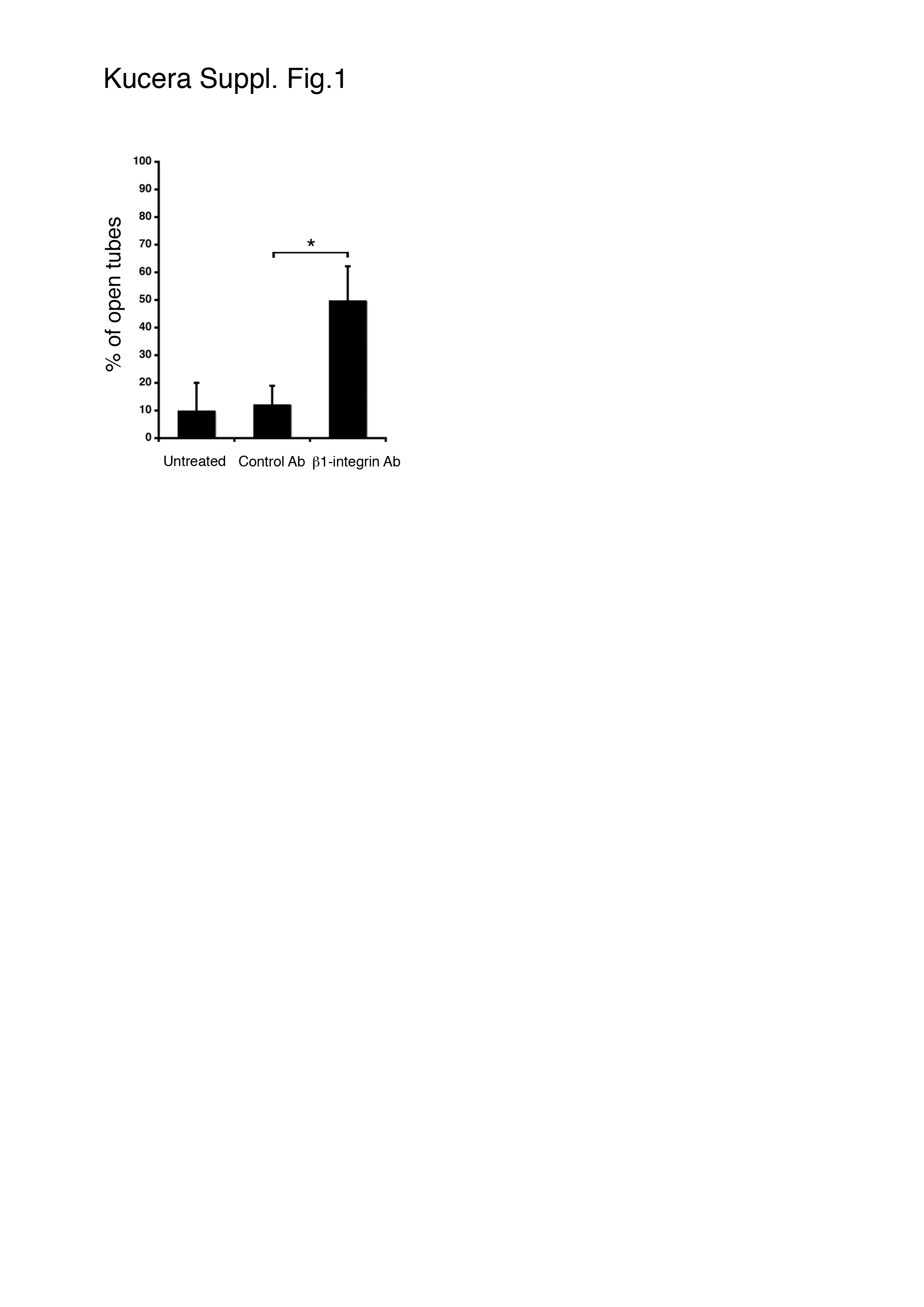

Supplement: Figure S1 — Impaired vascular tube formation of MS1 cells after treatment with β1-integrin blocking antibody. Quantification of open MS1 endothelial tubes in Matrigel overlay assays treated with control and β1-integrin blocking antibodies. N = 3 tube formation assays. *p<0.01. All values are means±SD. (0.60 MB TIF) [file pone.0004132.s001.tif]

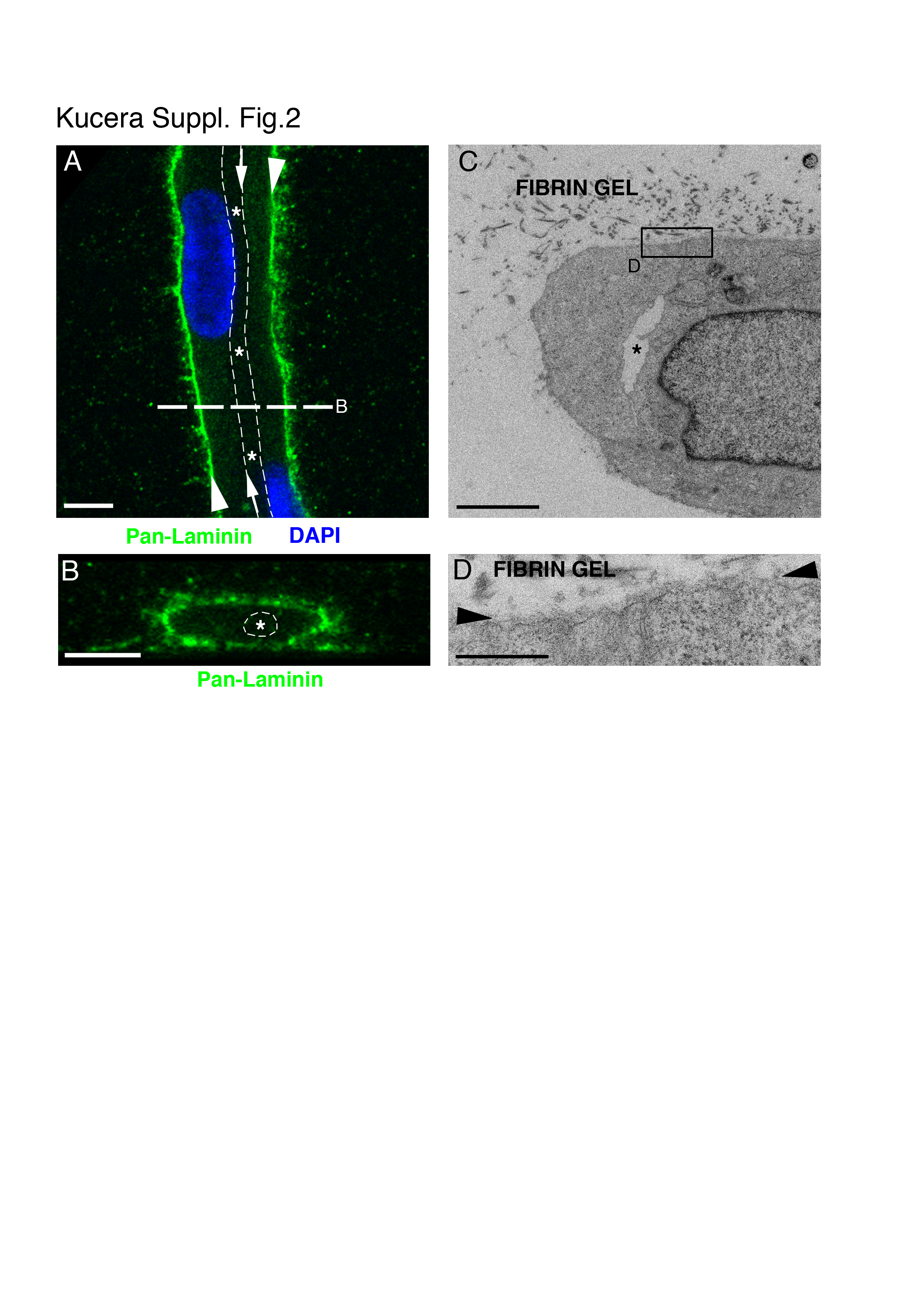

Supplement: Figure S2 — Localization of basement membrane and laminin in vascular tubes formed by HUVEC in fibrin gel. (A–B) Confocal images of a vascular tube formed by HUVEC in the fibrin gel stained for laminin (green). (A) A single plane of a z-stack shows laminin localized on the abluminal cell surface (arrowhead) facing the fibrin gel. The luminal plasma membrane (arrow) outlined by a dashed line surrounds the lumen (asterisk), which is free of laminin. Scale bar, 10 µm. (B) An optical cross-section is shown at the location indicated by a dashed line in (A). Nuclei (DAPI, blue). Scale bar, 10 µm. (C) Electron micrograph showing a cross-section through a vascular tube with a lumen (asterisk) formed by HUVEC in fibrin gel. Scale bar, 2 µm. (D) A higher magnification of a boxed area outlined in (C) showing the abluminal cell surface covered by a basement membrane (arrowheads). Scale bar, 500 nm. (4.70 MB TIF) [file pone.0004132.s002.tif]

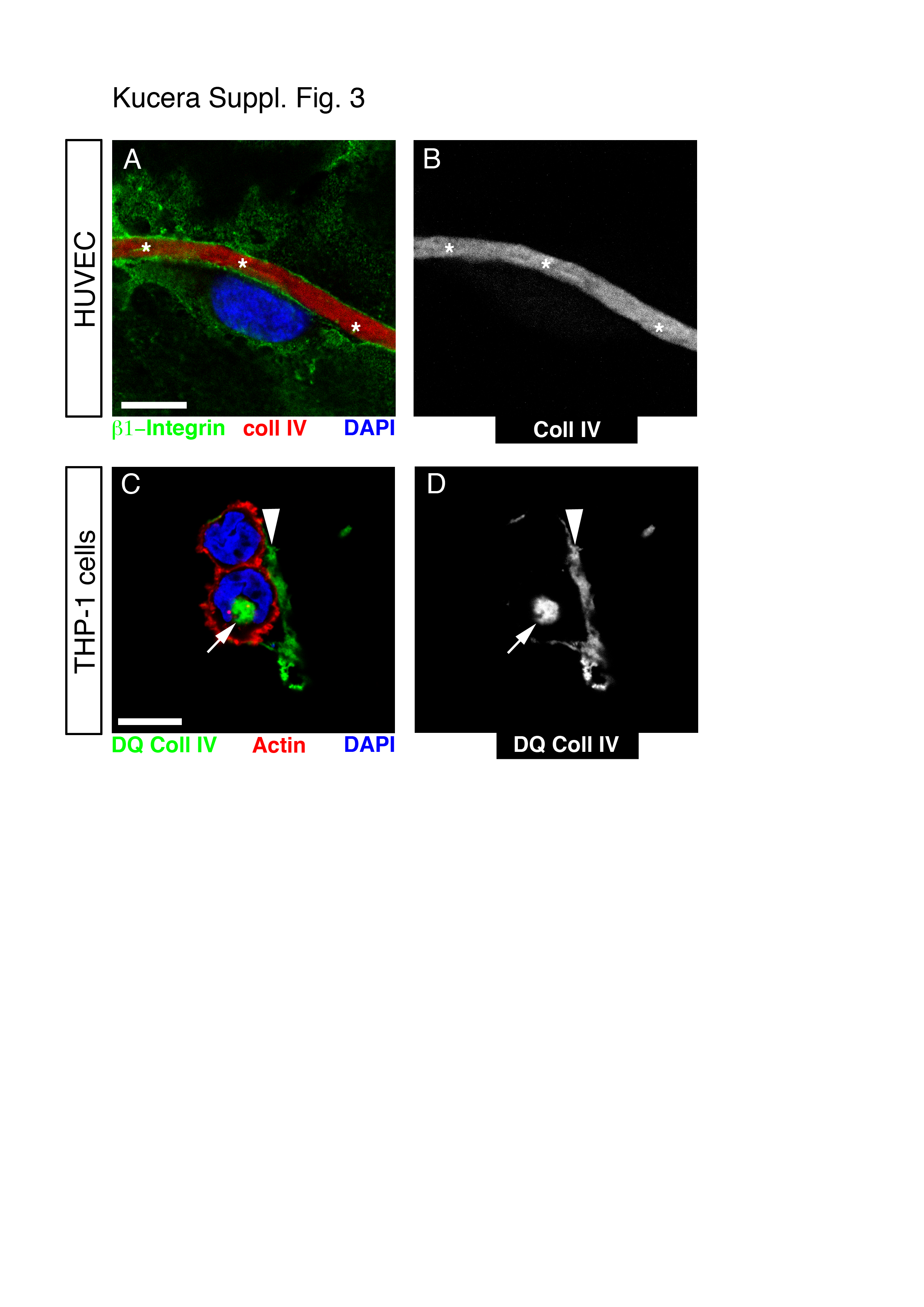

Supplement: Figure S3 — Mechanism of Matrigel removal by THP-1 macrophages. (A, B) Confocal images showing a vascular tube formed by HUVEC. (A) The tube is stained for collagen IV (red) and β1-integrin (green). Collagen IV fills the lumen (asterisk) of a tube. Nuclei (DAPI, blue). (B) The red channel from (A) shows the tube stained for collagen IV only. Scale bar, 10 µm. (C, D) Confocal images showing THP-1 cells in Matrigel mixed with DQ collagen IV. (C) The green channel fluorescence corresponds to the proteolytically degraded DQ collagen IV. THP-1 cells are stained for actin (red). The cleavage product can be found both intracellulary (arrow) and extracellulary (arrowhead) adjacent to THP-1 cell surfaces. Nuclei (DAPI, blue). (D) The green channel from (C) showing proteolytically cleaved DQ collagen IV only. Scale bar, 10 µm. (2.33 MB TIF) [file pone.0004132.s003.tif]
